# Chung’s swing technique: a new technique for small-incision lenticule extraction

**DOI:** 10.1186/s12886-016-0321-2

**Published:** 2016-09-01

**Authors:** Bu Ki Kim, Su Joung Mun, Dae Gyu Lee, Hyun Tae Choi, Young Taek Chung

**Affiliations:** Onnuri Smile Eye Clinic, Hyobong building 9F 1, Gangnam-daero 65 gil, Seocho-gu Seoul, Korea

**Keywords:** Small incision lenticule extraction, SMILE, Chung’s swing technique, Swing technique

## Abstract

**Background:**

To introduce the Chung’s swing technique for small-incision lenticule extraction (SMILE).

**Methods:**

A total of 112 eyes of 56 patients were included in this study. Patients were divided into two groups: 52 eyes of 26 patients were treated with SMILE using a traditional method (traditional group) and 60 eyes of 30 patients were treated with SMILE using the Chung’s swing technique (swing group).

**Results:**

At 1 month postoperatively, all eyes in both groups had an uncorrected distance visual acuity of 20/20 or better. The efficacy indices were 1.09 ± 0.17 and 1.02 ± 0.11 (*p* = 0.492), and the safety indices were 1.12 ± 0.14 and 1.09 ± 0.15 (*p* = 0.537), for the traditional and swing group, respectively. All eyes in both groups underwent successful lenticule extraction; all lenticules were intact and complete. The mean lenticule extraction times were 48.67 ± 4.9 and 39.8 ± 5.53 s, for the traditional and swing group, respectively (*p* < 0.001).

**Conclusions:**

The Chung’s swing technique is efficient for lenticule separation and extraction. Our study results showed good clinical outcomes.

**Trial registration:**

Trial registration number: KCT0001978. Registered 22 July 2016. Retrospectively registered.

**Electronic supplementary material:**

The online version of this article (doi:10.1186/s12886-016-0321-2) contains supplementary material, which is available to authorized users.

## Background

Small-incision lenticule extraction (SMILE) is a relatively new technique for the correction of myopia and myopic astigmatism, in which the corneal stromal lenticule is cut by a VisuMax® femtosecond laser (Carl Zeiss Meditec, Jena, Germany) and removed through a small (2–4 mm) corneal incision tunnel [1, 2]. Numerous studies on SMILE have reported excellent outcomes; thus, the technique has gained widespread acceptance due to its efficacy, predictability, and safety [1–4].

Although most of the operation is performed by a femtosecond laser, the manual steps (dissection and extraction of the lenticule) are crucial. However, locating the cap–lenticule and lenticule–stromal bed interfaces can be difficult, thereby extending the time required for the operation; additionally, the surgeon may have make multiple passes [5]. This can cause more corneal stromal damage, inflammation, and is prone to infection, so it can lead to delayed visual recovery, diffuse lamellar keratitis, corneal opacity, and poor visual outcomes.

In this study, we developed a simple, efficient method for lenticule separation during SMILE, referred to as the “Chung’s swing technique.” In this technique, after separating the lower interface of the lenticule, the dissector ascends to the upper interface by lifting and swinging the dissector at the upper margin of the lenticule. The Chung’s swing technique was compared with the traditional method, in terms of its efficacy, safety, and operation time.

## Methods

### Patients

This retrospective study recruited 112 eyes of 56 patients who underwent SMILE surgery to correct for myopia and/or myopic astigmatism at the Onnuri Eye Clinic in Jeonju, Korea from August 2014 to December 2014. Inclusion criteria for the study were as follows: myopia up to −10.0 D, astigmatism up to −4.0 D, a minimum age of 18 years, corrected distance visual acuity (CDVA) of 20/40 or better, and a minimum calculated postoperative residual stromal bed of 250 μm. Patients who had an accompanying ocular disease, prior history of ocular surgery, or any contraindication to refractive surgery were not included. Patients were randomly divided into two groups: the traditional method group (traditional group) and the Chung’s swing technique group (swing group). The study was approved by the Internal Regulatory Board of Yeouido St. Mary’s Hospital, Seoul, Republic of Korea (SCI15RISI0130). Written informed consent for study participation was obtained from all participants.

Patients underwent preoperative examination, which included measurement of their uncorrected distance visual acuity (UDVA), CDVA, manifest and cycloplegic refraction, and intraocular pressure via tonometry (CT-80, Topcon, Tokyo, Japan). Patients also underwent slit-lamp microscopic examination, fundus examination, autokeratometry (KR-8900, Topcon), specular microscopy (noncom Robo-ca, Konan Medical, Hyogo, Japan), topography (Orbscan® IIz, Bausch & Lomb, Rochester, NY, USA), and corneal thickness measurements (Galilei, Ziemer Ophthalmic Systems, Port, Switzerland).

### Surgical technique

The same surgeon (CYT) performed all surgical procedures. A VisuMax® 500-kHz femtosecond laser was used for SMILE treatment (frequency: 500 kHz; cut energy index: 180 nJ pulsed; spot spacing: 4.5 μm). The lenticule diameter was 6.5 mm and the cap diameter was 7.5 mm. The intended thickness of the cap was 110 μm, and the incision was 2.0 mm long at the 11 o’clock position. The lenticule was separated using a straight, blunt spatula. The traditional method has been described previously [2].

**Fig. 1:**
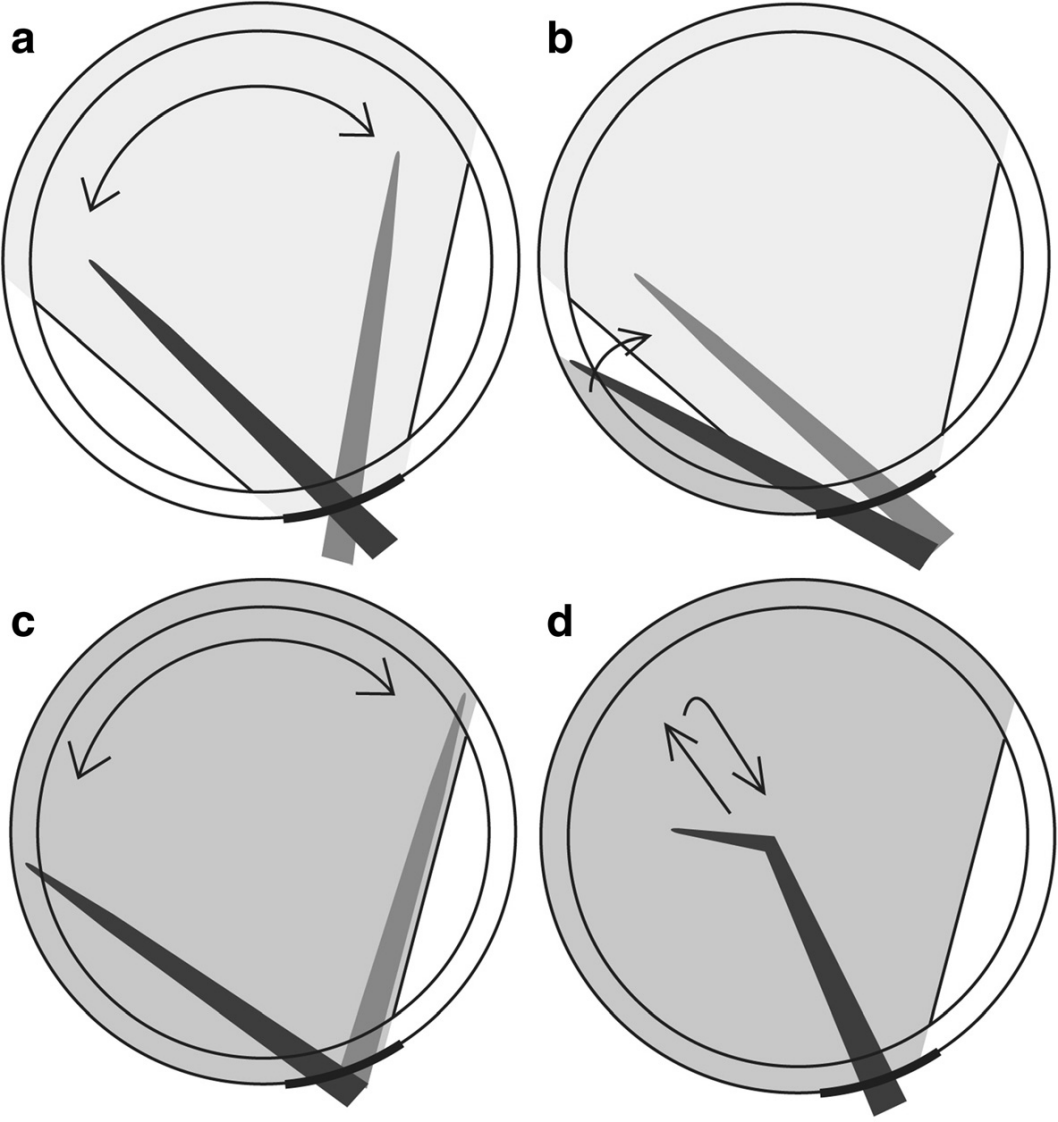
Diagram of the steps of the Chung’s swing technique. **a** The lenticule–stroma interface was dissected in a fan-shape using a spatula. **b** The spatula ascended to the lenticule–cap interface by lifting and swinging at the left end of the incision. **c** The lenticule–cap interface was dissected in the same way. **d** The lenticule was extracted by McPherson forceps. After grasping the lenticule margin at 12 o’clock, the lenticule was pushed and pulled to the center of the cornea

**Fig. 2:**
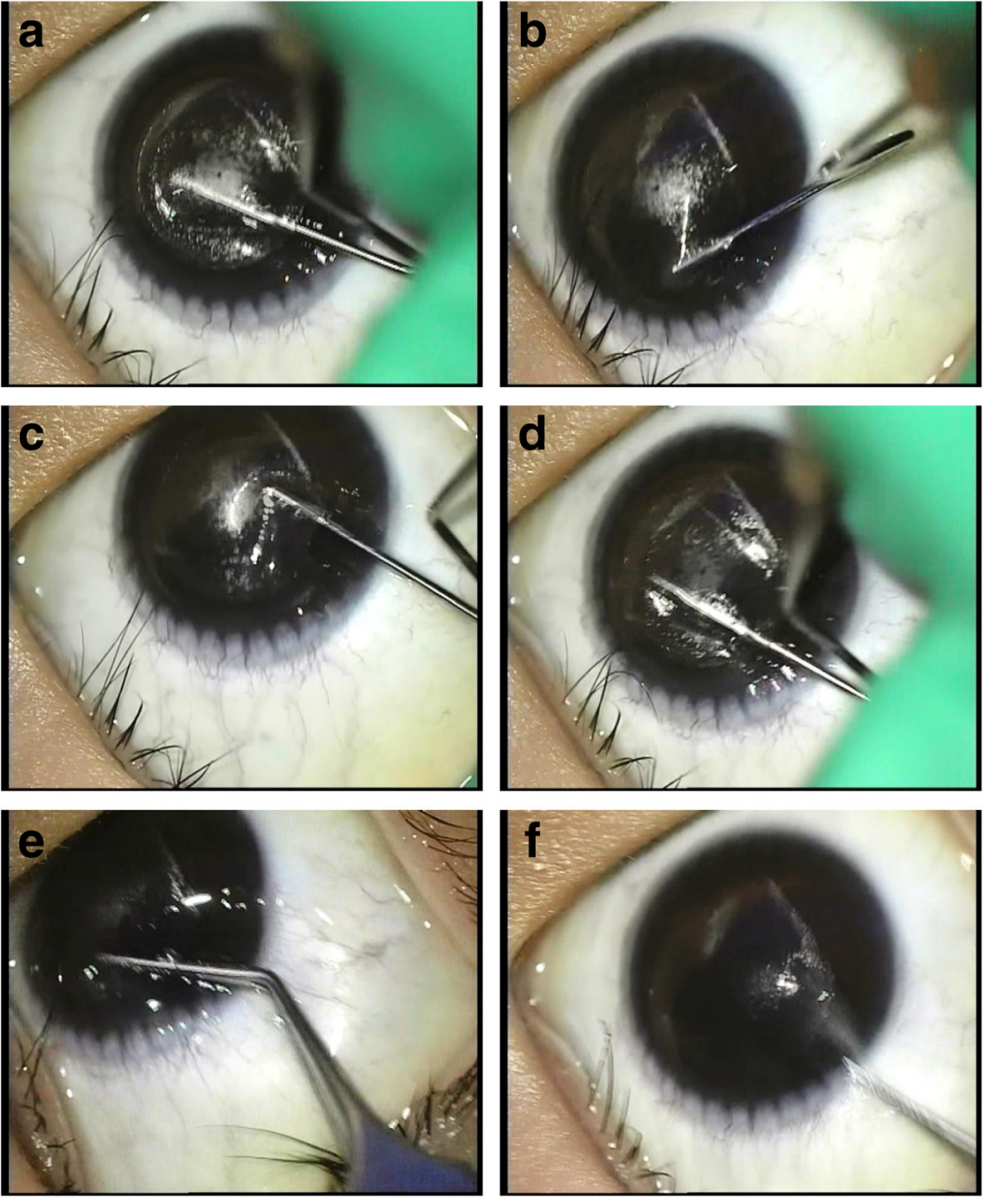
Intraoperative photographs of the Chung’s swing technique. **a** Dissection of the lenticule–stroma interface in a fan-shape. **b** Ascending the spatula to the lenticule–cap interface. **c**, **d** Dissection of the lenticule–cap interface by swinging the spatula. **e** Pushing the forceps anteriorly with strength. **f** Pulling the forceps posteriorly with strength

The Chung’s swing technique procedure is described in the following. The lenticule–stromal bed interface (i.e., the lower lenticule interface) is separated with a fan-shaped spatula,without grasping the conjunctiva with forceps. The spatula ascends to the lenticule-cap interface by lifting and swinging at the left end of the incision. After the lenticule-cap was separated into a fan shape, McPherson forceps (M. Blum design; Geuder, Heidelberg, Germany) grasp the lenticule margin at 12 o’clock; the lenticule is pushed towards the center of the cornea and pulled, to remove the lenticule in a clockwise direction. Because there is mild resistance at the 12–3 o’clock positions and 8–11 o’clock positions for lenticule movement and removal, push and pull of the lenticule requires some effort (Figs. 1 and 2) (Additional file 1). Both the lenticule-stromal bed and the lenticule-cap interface were completely separated, except at the 12 o’clock to 3 o’clock and the 8 o’clock to 11 o’clock positions, to avoid damage by forceps during lenticule extraction. In the case of a ripped lenticule, McPherson forceps were inserted again to remove the lenticule remnant.

After removing the lenticule, the stromal pocket was flushed with balanced salt solution (BSS®, Alcon, Fort Worth, TX, USA). After surgery, all patients were treated with 0.5 % moxifloxacin (Vigamox®, Alcon) for 5 days, a 0.1 % fluorometholone (Ocumetholone®, Samil, Seoul, Korea) for 2 weeks, and preservative-free hyaluronic acid lubricating drops (Hyalein Mini 0.1 %®, Santen, Osaka, Japan) for at least 2 weeks.

### Outcome measures

The lenticule was examined with a microscope to check its integrity as soon as it was extracted; the duration of the lenticule extraction was recorded on video. The patients were evaluated at 1 day, 1 week, and 1 month after surgery. At each visit, the UDVA and CDVA were measured; a slit-lamp exam was performed, in addition to manifest refraction and corneal topography measurements, and the complications were assessed. The patients’ satisfaction was evaluated at postoperative day 1 based on the patients’ complaints.

### Statistical analyses

Statistical analyses were performed using the SPSS for Windows software package (ver. 18.0; SPSS Inc., Chicago, IL, USA). Graphics were generated using the Microsoft Excel 2013 software (Microsoft Corporation, Redmond, WA, USA). All data are given as means ± standard deviation. Statistical analyses of visual acuity used the logarithm of the minimum angle of resolution (logMAR). Student’s *t*-test was used to compare the two groups, and the Mann–Whitney *U*-test was used to compare the length of time for lenticule extraction between the two groups. A *p*-value < 0.05 was considered to indicate statistical significance.

## Results

### Study population

A total of 52 and 60 eyes were included in the traditional and swing groups, respectively. The target refraction was emmetropia (±0.25 D) in all eyes. The mean age of participants in the traditional and swing groups was 26.28 ± 6.63 and 24.08 ± 5.42 years, respectively. The mean preoperative spherical equivalent was −5.69 ± 1.53 D and −5.07 ± 1.80 D for the traditional and swing groups, respectively (*p* = 0.656). There was no significant group difference with respect to corneal power, UDVA, CDVA, or corneal thickness (Table 1).

**Table 1:** Patient demographics

Characteristics	Traditional group	Swing group	*p*-value
Eyes (n)	52	60	
Sex (M/F)	16/36	22/38	
Age (years)	26.28 ± 6.63 (18 ~ 39)	24.08 ± 5.42 (18 ~ 36)	0.127
Mean corneal power (dioter)	43.99 ± 1.6 (40 ~ 47.75)	44.27 ± 1.58 (39.75 ~ 49.25)	0.451
UDVA (logMAR)	1.17 ± 0.47 (0.7 ~ 2)	1.15 ± 0.49 (0.4 ~ 2)	0.996
CDVA (logMAR)	−0.04 ± 0.05 (−0.1 ~ 0.1)	−0.05 ± 0.05 (−0.1 ~ 0.1)	0.897
IOP (mmHg)	15.7 ± 2.94 (9 ~ 19)	14.5 ± 2.42 (11 ~ 18)	0.781
Sphere (diopter)	−5.19 ± 1.49 (−8.75 ~ −2.75)	−4.52 ± 1.79 (−9.0 ~ −3.5)	0.674
Cylinder (diopter)	−1.01 ± 0.43 (−2 ~ 0)	−1.10 ± 0.44 (−2 ~ −0.5)	0.718
Spherical equivalent (diopter)	−5.69 ± 1.53 (−9.13 ~ −2.75)	−5.07 ± 1.80 (−9.25 ~ −3.38)	0.656
Central corneal thickness (μm)	522.8 ± 28.7 (481 ~ 571)	513.4 ± 30.4 (488 ~ 580)	0.524
Expected residual corneal bed (μm)	319.6 ± 29.1 (254 ~ 364)	308.7 ± 28.8 (252 ~ 351)	0.447

### Efficacy

All 112 eyes were examined at 1 day, 1 week, and 1 month postoperatively. At 1 day, the UDVA was higher for the swing group (−0.02 ± 0.05) than for the traditional group (0.01 ± 0.04); but the difference was not significant (*p* = 0.507). No significant group differences were observed regarding postoperative UDVA or CDVA during the follow-up period (Table 2). At 1 day, 1 week, and 1 month, 70.8 %, 87.5 %, and 100 % of eyes in the traditional group, respectively, and 83.3 %, 91.7 %, and 100 % of eyes in the swing group, respectively, had a UDVA of 20/20 or better.

**Table 2:** Group comparison of uncorrected and corrected distance visual acuity

		POD		
		1 day	1 week	1 month
UDVA (logMAR)	Traditional group	0.01 ± 0.04	−0.04 ± 0.05	−0.08 ± 0.08
	Swing group	−0.02 ± 0.05	−0.04 ± 0.05	−0.07 ± 0.07
	*p*-value	0.507	0.728	0.836
CDVA (logMAR)	Traditional group	−0.01 ± 0.08	−0.07 ± 0.05	−0.08 ± 0.06
	Swing group	−0.02 ± 0.07	−0.06 ± 0.07	−0.07 ± 0.06
	*p*-value	0.981	0.527	0.764

The efficacy indices (mean postoperative UDVA / mean preoperative CDVA at 1 month) were 1.09 ± 0.17 and 1.02 ± 0.11 for the traditional and swing groups, respectively. There was no significant difference between the two groups (*p* = 0.492).

### Safety

**Fig. 3:**
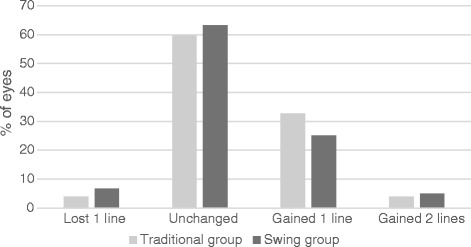
Safety comparison of SMILE techniques. CDVA gain and loss at 1 month postoperatively

Figure 3 shows CDVA gains and losses at 1 month postoperatively. In the traditional group, 3.85 % had lost one line of CDVA, 59.62 % had an unchanged CDVA, 32.69 % had gained one line, and 3.85 % had gained two lines. In the swing group, 6.67 % had lost one line of CDVA, 63.33 % had an unchanged CDVA, 25.0 % had gained one line, and 5.0 % had gained two lines. No eyes lost two lines of CDVA in either group.

The safety indices (mean postoperative CDVA at 1 month / preoperative CDVA) were 1.12 ± 0.14 and 1.09 ± 0.15, for the traditional and swing groups, respectively. There was no significant difference between the two groups (*p* = 0.537).

### Predictability

The mean postoperative spherical equivalent values were −0.07 ± 0.39 D, −0.06 ± 0.37 D, and −0.08 ± 0.36 D for the traditional group, and −0.03 ± 0.38 D, −0.03 ± 0.42 D, and −0.04 ± 0.41 D for the swing group at 1 day, 1 week, and 1 month, respectively. No significant group differences were evident in the postoperative spherical equivalent for all follow-up visits. In the traditional group, 90.38 % and 100 % were within ±0.5 D and ±1.0 D of the intended correction at 1 month postoperatively, respectively. In the swing group, 88.33 % and 98.33 % were within ±0.5 D and ±1.0 D of the intended correction at 1 month, respectively.

### Lenticule integrity

All eyes in both groups showed successful lenticule extraction. There was no case of lenticule tearing. All lenticules had intact round margins in both the traditional and swing groups.

### Duration of lenticule extraction

The mean lenticule extraction time was 48.67 ± 4.9 and 39.8 ± 5.53 s for the traditional and swing groups, respectively. For the swing group, the time required for lenticule extraction was significantly shorter than that of the traditional group (*p* < 0.001).

### Patient satisfaction

Regarding complaints, 23.1 % of the traditional group and 20.0 % of the swing group complained of foreign body sensations or pain, 46.2 % of the traditional group and 33.3 % of the swing group complained of blurred vision, and 23.1 % of the traditional group and 10.0 % of the swing group complained about the postoperative day 1 injection.

### Complications

Incisional edge tearing during separation of the lenticule was observed in two cases in the traditional group; however, there was no incisional edge tearing in the swing group.

Corneal trace haze was observed in 5.7 % of the traditional group and 3.3 % of the swing group; however, this complication did not appear to have an effect on visual acuity. No complications (corneal opacity, epithelial ingrowth, keratitis, or ectasia) were observed during the follow-up period in either group.

## Discussion

Although small incisions in SMILE surgeries provide patients with the advantages of greater biomechanical strength and less severe denervation of the cornea, the SMILE procedure requires greater surgical skill compared with other refractive surgeries, because the lenticule is manually removed through the small incision [6–8]. Surgeons may have difficulty separating the lenticule; thus, the separation procedure may require several attempts. This results in longer surgeries; additionally, these difficulties increase the likelihood of diffuse lamellar keratitis or even surgical failure [5, 9]. During separation or extraction of the lenticule, a torn incision edge can increase denervation of the cornea, resulting in pain and foreign body sensation.

The SMILE procedure consists of a series of laser and manual steps. In the traditional method, following lenticule formation via a VisuMax^®^ (Carl Zeiss Meditec) femtosecond laser, the lenticule is extracted as follows: (1) The anterior surface of the lenticule is separated from the overlying cornea using a blunt spatula. (2) A small, pointed spatula is used to enter the cleavage plane on the posterior side of the lenticule to separate the edge of the lenticule. (3) The blunt spatula is used to separate the posterior surface of the lenticule from the underlying stroma. (4) Microforceps grasp the lenticule and extract it through the incision.^2^

Recently Zhao et al. [5] introduced the continuous curvilinear lenticulerrhexis (CCL) technique, which merges steps 3 and 4 into one step, by tearing and extracting the lenticule from the stromal bed without separation in a continuous, circumferential manner. They reported that eyes treated by SMILE using the CCL technique showed favorable results in terms of efficacy, safety, operation time, and lenticule margin integrity. Furthermore, the CCL technique tended to reduce disturbance to the corneal tissue by instruments, and shortened the learning curve required for lenticule extraction thereby allowing surgeons to perform SMILE more safely and efficiently [5]. However, the CCL technique has several limitations. During the CCL procedure, the lenticule can become ripped, especially in cases of mild myopia in which lenticule extraction is more difficult. In this case, the operation times are longer and the number of removal attempts is higher. Note that Zhao et al. [5] did not include low-degree myopia in their study. Also, the CCL technique requires separation of the edge of the lenticule so that it can be grasped by microforceps; this step is one of the most difficult parts of lenticule extraction, especially for less experienced surgeons. Furthermore, Zhao et al. [5] recommended to not use the CCL technique in the presence of an opaque bubble layer (OBL), uneven laser scanning, or in any other situation that may increase the difficulty of lenticule extraction. Although there have been no studies on the incidence of OBL formation during SMILE surgeries, Liu et al. [10] reported an OBL rate of 52.5 % during femtosecond laser-assisted *in situ* keratomileusis (LASIK) procedures, which represents a considerable effect.

The Chung’s swing technique that we developed is simple and fast. The key advantage of the Chung’s swing technique is that the surgeon does not need to find both the anterior and posterior interfaces of the lenticule edge near the incision. Once the posterior interface of the lenticule is identified and separated, the anterior interface of the lenticule can be easily found by lifting and swinging the spatula tip at the left end of the incision. In the current study, we reduced the operation time using this technique; the lenticule extraction time was significantly shorter in the swing group than in the traditional group. Therefore the UDVA at 1 day postoperatively was expected to be better in the swing group than in the traditional group and fewer patients in the swing group complained of blurred vision compared to the traditional group; however, no significant group difference in the UDVA was observed. We attributed this to the fact that all surgeries were performed by one skillful surgeon; thus, the time required for lenticule separation and extraction was sufficiently short as to prevent postoperative corneal edema.

In this study, lenticule extraction was successful for all eyes in both groups, with all lenticules intact and complete; also, the UDVA was ≥ 20/20 at 1 month postoperatively. In the traditional and swing groups, 90.38 % and 88.33 %, respectively, were within ±0.5 D of the intended correction at 1 month postoperatively. These results are consistent with previous studies [1–4]; no significant difference was observed between the traditional and swing groups. This suggests that the Chung’s swing technique is as efficient as the traditional method; furthermore, lenticule extraction with the Chung’s swing technique is faster than that of the traditional method, making it more convenient.

In terms of complications, there were two cases of incisional edge tearing in the traditional group. According to Moshirfar et al. [11] incisional edge tearing occurred in 1.5 % of eyes during the SMILE procedure. In this study, 3.8 % of eyes in the traditional group, and no eyes in the swing group, experienced incisional edge tearing; however, in these two cases, the tearings were < 0.3 mm; the patients did not complain of pain or foreign body sensation postoperatively. In the Chung’s swing technique, the 12–3 o’clock and 8–11 o’clock positions were left unseparated during dissection, as opposed to the traditional method in which the lenticule is separated completely. In the Chung’s swing technique, the lenticule is easily extracted by pushing and pulling with forceps, and incisional edge tearing is less likely. There were concerns about edge tearing when the spatula ascended to the lenticule-cap interface by lifting and swinging; however, only the tip of the spatula was lifted to avoid incisional edge stress. Because there was mild resistance at the 12 o’clock to 3 o’clock and 8 o’clock to 11 o’clock positions, the lenticule could be ripped during extraction. In this event, the forceps could be reinserted to remove remnants of the lenticule, which might result in longer surgical times or diffuse lamellar keratitis. However, there were no ripped lenticules, so there were no differences in resistance during lenticule extraction between the groups. Moreover, in the traditional method, we had to grasp the conjunctiva by forceps near the limbus during lenticule separation, whereas we did not grasp the conjunctiva in the swing method. This prevented postoperative pain and injection, and there were fewer patients who complained of pain and the injection at postoperative day 1 in the swing group.

This study had several limitations, including a retrospective rather than prospective design: the results would have been more reliable if we had designed a prospective study such that one eye had SMILE surgery using the traditional method and the other eye had SMILE with the swing method. Confocal microscopy or histologic examination would be helpful to further evaluate the technique.

## Conclusions

The Chung’s swing technique is a new method of lenticule separation and extraction in the SMILE procedure. This simple, fast approach allows surgeons to easily separate and extract the lenticule, with minimal incisional edge tearing; additionally, the technique is not limited by the degree of myopia or corneal conditions, such as OBL. We expect that this technique will be preferred by surgeons performing SMILE.

## Abbreviations

CCL, continuous curvilinear lenticulerrhexis; CDVA, corrected distance visual acuity; LASIK, laser-assisted *in situ* keratomileusis; logMAR, logarithm of the minimum angle of resolution; SMILE, small incision lenticule extraction; UDVA, uncorrected distance visual acuity

## Additional file

Additional file 1: Shows the SMILE procedure using the Chung’s swing technique. (MP4 5314 kb)
